# Developing Rapid Urine-Penetrating, Self-Drying, and Breathable Materials for Disposal Diapers

**DOI:** 10.3390/polym18192383

**Published:** 2026-09-29

**Authors:** Yuanduo Wang, Dan Wang, Yuxiao Wang, Mengyao Ruan, Xiangyu Jin, Andreii S. Kritchenkov, Gilson Khang, Sergei V. Kostjuk, Jiansheng Guo, Wanjun Liu

**Affiliations:** 1 Shanghai Frontiers Science Center of Advanced Textiles, College of Textiles, Donghua University, Shanghai 201620, China; 2 Department of Inorganic Chemistry, Peoples’ Friendship University of Russia, Moscow 117198, Russia; 3 Institute of Technical Acoustics NAS of Belarus, 210009 Vitebsk, Belarus; 4 Department of Bionanotechnology and Bio-Convergence Engineering, Jeonbuk National University, Jeonbuk 54896, Republic of Korea; 5 Department of Chemistry, Belarusian State University, 220006 Minsk, Belarus; 6 Institut Parisien de Chimie Moleculaire, Centre National de la Recherche Scientifique, Sorbonne Universite, 75252 Paris, France

**Keywords:** diapers, meshed pores, urine penetration, self-drying

## Abstract

Disposable diapers are an essential component of infant and incontinent individual care, providing superior urine absorption. However, achieving a key goal for high-performance diapers is rapid urine penetration concurrent with surface self-drying. This assertion is substantiated by the prevalence of diaper dermatitis, which afflicts 29% of affected individuals. In this study, we designed rapid urine-penetrating, self-drying materials (RIPPING materials) by the synergy between the meshed pore hydrophobic top layer and superabsorbent middle layer. It is noteworthy that the meshed pores of the hydrophobic top layer facilitated expeditious urine absorption by the subjacent superabsorbent middle layer (0 g urine run-off). Furthermore, the urine would be retained on the surface of the diaper by the mesh, due to the material’s inherent hydrophobic properties. Following the systematic optimization of the structure of RIPPING materials, superior self-drying diapers with a wetness of 0.06 ± 0.01 g were achieved after urine absorption by preventing the direct contact between the skin and superabsorbent materials. The breathability of disposable diapers is attributed to the use of breathable materials, while its high-performance back layer exhibits efficacy in resisting hydrostatic pressure. Consequently, the present study proposes a high-performance RIPPING diaper that simultaneously achieves outstanding breathability without compromising leak resistance.

## 1. Introduction

Disposable diapers are a urine absorption system to maintain a relatively dry skin and sanitary environment, which has been widely used for infants and incontinent individuals [1,2,3]. For example, more than 6000 diapers are needed for each infant [4]. However, the prevalence rate of diaper dermatitis remains as high as 29% [5,6], although being significantly reduced by employing high-performance diapers. Notably, diaper dermatitis results in 25% of acute care visits of neonates and infants [7]. In general, disposable diapers are usually designed with a laminated structure containing a top layer, an acquisition layer, a superabsorbent middle layer, and a back layer [8,9,10]. The top layer can transmit urine into the superabsorbent layer quickly and keep the diaper surface dry at the same time [11], the superabsorbent middle layer can absorb and store urine, while the back layer can prevent cloth contamination from urine leakage [12]. In addition, a soft surface and high breathability are highly desired for diapers to improve the comfortability of wearing.

Up to now, considerable efforts have been devoted to enhancing the properties of disposable diapers in order to fight diaper dermatitis [2,13,14,15,16]. A key point is to accelerate the urine penetration from the top layer to the superabsorbent layer so that less urine will stay at the interface between the diaper and skin. To date, porous nonwoven materials with a hydrophilic surface are usually used as the top layer of diapers. The most frequently used top layer materials are through-air bonded nonwovens made of polypropylene (PP)–polyethylene (PE) core–shell fibers that are generally treated with hydrophilic finishing. Meanwhile, medical-grade petroleum jelly or ointment can also be applied to the top layer of diapers, which acts as a barrier of skin conditioner to moisture and irritation [17,18]. Nevertheless, the hydrophilic surface becomes wet after absorbing urine and is seldom able to maintain the diaper surface dry because of wettability itself, which not only results in a sense of discomfort [19], but also leads to diaper dermatitis due to excessive hydration of the stratum corneum, making the skin more vulnerable to frictional force and irritation from urine [20,21,22]. One potential solution is to develop high-performance materials for the top layer based on one-way water-transport materials [23,24,25].

Another important design for diapers is the use of an acquisition layer placed between the top layer and the superabsorbent layer, which can help distribute urine and increase the absorption surface area of the superabsorbent layer. In this way, the urine absorption capacity of the superabsorbent layer can be maximized by preventing the rapid saturation of urine absorption in the topical spot [26,27]. However, the employment of an acquisition layer will inevitably slow down the urine penetration speed and result in more unabsorbed urine on the diaper surface. In addition, the prevalence and severity of diaper dermatitis have been mitigated by improving the breathability of diapers using a microporous membrane as the back layer [20,28]. Such a breathable back layer is usually made of a waterproof polyethylene film with tiny holes so that water vapor cannot pass through while urine leakage can be prevented at the same time [29]. To this end, it remains a huge challenge to develop breathable diapers that can efficiently absorb urine and maintain a superior dry surface so that the prevalence rate of diaper dermatitis can be further decreased, although the diaper performance has been greatly elevated by innovating on the design of diaper structures and material properties.

In this study, we developed highly breathable materials capable of transporting urine quickly and maintaining a dry surface at the same time. These functions were achieved by combining a meshed pore hydrophobic material as the top layer, a superabsorbent material as the middle layer, and a breathable and hydrophobic material as the back layer. In particular, the combination of the superabsorbent middle layer and the hydrophobic top layer with meshed pores allowed rapid urine penetration and minimal urine wetback simultaneously. By systematically investigating the properties of the top, middle, and back layers, and their combinations, especially the influence of meshed pore structure on urine transportation, and self-drying properties, high-performance materials for the use of diapers were obtained, which was verified by comparing with the most popular Merries diapers.

## 2. Materials and Methods

### 2.1. Chemicals and Materials

Sodium chloride (99%), calcium chloride, magnesium sulfate heptahydrate, and carbamide were obtained from Sigma-Aldrich (St. Louis, MO, USA). Superabsorbent materials containing cross-linked sodium polyacrylate particles (SAPs) were kindly provided by Hangzhou Haoyue Industrial Co., Ltd. (Hangzhou, China). Notably, the superabsorbent materials exhibit a sandwich structure. In the middle layer, SAPs are dispersed and immobilized by PE/PP bi-component fibers. Additionally, the middle layer is further covered by air-laid nonwovens composed of wood pulp and ES fibers that further provide physical confinement and prevent leakage of SAPs. Polypropylene melt-blown materials were kindly provided by Xuancheng Guangneng Nonwoven Co., Ltd. (Xuancheng, China). Commercial diapers were purchased from Kao Group (Merries, Shanghai, China).

### 2.2. Fabrication of Rapid Urine-Penetrating, Self-Drying, and Breathable Materials (RIPPING Materials)

Polypropylene melt-blown materials were used as the top layer and the back layer. It should be noted that the polypropylene melt-blown materials were further processed using a laser engraving machine (FD-460, Kerui Laser Machinery Co., Ltd., Liaocheng, China) to obtain a meshed pore structure. To ensure complete cutting of meshed pores and get rid of deformation during engraving, optimized parameters of cutting speed and peak pulse power were kept constant at 90 mm s^−1^ and 8 W, respectively. The RIPPING diapers were achieved through thermal bonding of the hydrophobic top layer, superabsorbent middle materials, and the hydrophobic back layer, using a thermal bonding machine (550, Changzhou Wujin Guangyu Embossing Roller Machinery Co., Ltd., Changzhou, China). The bonding temperature, bonding speed, and bonding pressure were kept constant at 157 °C, 1 m min^−1^, and 0 MPa, respectively.

### 2.3. Characterization

Scanning electron microscopy (SEM, TM 3000, Hitachi, Ltd., Tokyo, Japan) was used to observe the morphology of the materials. Images were taken at an acceleration voltage of 15.0 kV. Contact angles were measured using a contact angle goniometer (OCA15EC, Data Physics Corporation, Stuttgart, Germany) by adding a small droplet (5 μL) of distilled water. The pore size distribution was obtained through a PMI aperture tester (CFP-1100-AI, Porous Materials Inc. Ithaca, NY, USA) through wet up/dry up method, using SilWick with a surface tension of 20.1 dynes cm^−1^ and the sample diameter was around 18.3 cm. Thickness was measured using a fabric micrometer (YG141N, Nantong Hongda Experiment Instrument CO., Ltd., Nantong, China) according to ISO 9073-2: 1995, MOD Standard [30], and the applied force and the area of presser foot were 125 cN and 2500 mm^2^, respectively.

Air permeability. Air permeability was examined using an Auto Air Permeability Tester (YG461E, Guoliang Yiqi Co., Ltd., Wuhan, China) according to the ISO 9073-2009 Standard, and the sample size and pressure drop were 20 cm^2^ and 200 Pa, respectively. Mechanical properties were examined by a multi-function electronic fabric strength machine (YG026MB, Fangyuan Instrument CO., Ltd., Wenzhou, China) according to the ISO 9073-3: 1989 Standard. The sample size, stretching rate, and gage length were 280 mm × 50 mm, 100 ± 10 mm min^−1^, and 200 mm, respectively. Samples were pre-conditioned for at least 1 day (relative humidity = 65%, temperature = 20 °C).

Preparation of synthetic urine. Synthetic urine was prepared according to the reported method [31]. Briefly, carbamide (0.325 mol L^−1^), sodium chloride (0.137 mol L^−1^), magnesium sulfate heptahydrate (0.004 mol L^−1^), and calcium chloride (0.005 mol L^−1^) were dissolved in distilled water.

Urine absorption capacity. To quantify the urine absorption capacity of superabsorbent materials, samples with a size of 100 mm × 100 mm were dipped into 1000 mL urine and weighed before and after absorbing urine. The urine absorption capacity was determined by the following Equation (1):

Q = (m_2_ − m_1_)/m_1_, (1)

where Q is urine absorption; m_1_ and m_2_ are the weight of samples before and after absorbing urine. It should be noted that urine absorption as a function of time was tested under no pressure, while saturated urine absorption as a function of pressure was obtained by 1 h urine absorption under different pressures.

Urine retention rate. To measure the urine retention rate, samples with a size of 100 mm × 100 mm were submerged in urine for 1 h under no pressure, and then the saturated samples were weighed at different time points after being placed on a desk in ambient conditions (relative humidity = 65%, temperature = 20 °C). The urine retention rate was determined by the following Equation (2):

Y = m_n_/m_0_*100%, (2)

where Y is the urine retention rate; m_0_ is the wet weight right after urine absorption; m_n_ is the wet weight after putting the saturated samples in ambient conditions for a specific time.

Strike-through time (STT). The repeated urine STT was tested using a liquid penetration instrument (YG814D, Fangyuan Instrument Co., Ltd., Wenzhou, China) according to the ISO 9073-13: 2006 Standard. The key element to determine STT was a strike-through plate which was a 25 mm thick transparent acrylic sheet with a mass of 500 ± 5 g, fitted with corrosion-resistant electrodes consisting of 1.6 mm diameter platinum wire set in grooves of the cross-section 4.0 mm × 7.0 mm cut in the base of the plate. The strike-through plate was fixed in a baseplate (the surface area was 100 mm × 100 mm) which will contact the tested sample. The tested sample was put right below the baseplate, and the distance between the electrodes and the sample was 4 mm. During the experiment, the initial flow of urine liquid will complete the electrical circuit and start the timer. The timer will stop when the urine liquid has penetrated tested samples and fallen below the level of the electrodes in the strike-through plate. According to the ISO 9073-13: 2006 Standard, the tested sample with a size of 100 mm × 100 mm was put on top of an eight-layer standard absorption medium (spunlaced nonwovens, STT was about 1.7 s), and synthetic urine was poured through a funnel which was 5 ± 0.5 mm above the top of the electrodes 3 times (5 mL each time, 1 min interval); thus, 3 different STT were obtained.

Run-off. This test was conducted according to the Textiles-Test methods for nonwovens-Part 11: Run-off (ISO 9073-11:2006). The value of run-off was evaluated by the mass difference in the standard absorption medium. In brief, 40 mL urine (RIPPING materials) was discharged at a prescribed rate (6.25 mL s^−1^) onto a test specimen (100 mm × 300 mm) which was superimposed on a standard absorbent medium and placed on an inclined table with an angle of 10°. All excess urine that ran down the test specimen was collected by a standard absorption medium placed below the lower end of the test specimen. The run-off was calculated by using Equation (3):

Y = M_A_ − M_B_, (3)

where M_B_ and M_A_ stand for the mass of standard absorption medium (g) before and after absorbing the excessive urine.

Urine remaining, urine wetback, and wetness. These tests were custom-defined to understand the influence of meshed pore structure on the self-drying property of the RIPPING materials. In brief, urine remaining, urine wetback, and wetness were defined as the mass of residual urine on the surface of materials after absorbing urine, the mass of urine wetback from the superabsorbent through the meshed pores of the top layer, and the overall surface wetness of the RIPPING materials after absorbing urine, respectively.

Specifically, the urine remaining of the meshed pore top layer was obtained by evenly pouring synthetic urine onto the specimen (100 mm × 100 mm) 3 times (5 mL each time, 1 min intervals). It should be noted that the specimen was held in the air to allow urine to penetrate through the meshed pores without any influences. After the third pouring of urine, a standard absorption medium (m_1_) was placed on the specimen and pressed by briquetting (diameter = 100 mm, weight = 4 kg). After 1 min of absorption, the mass of the standard absorption medium was determined (m_2_). Therefore, the urine remaining of the meshed pore top layer was characterized by the mass difference in the standard absorption medium (m_2_-m_1_). While the urine remaining of RIPPING materials was tested in a similar method, the RIPPING materials were placed on an eight-layer standard absorption medium (spunlaced nonwovens, STT was about 1.7 s). After the third pouring of urine, the superabsorbent middle layer of the RIPPING materials and the standard absorption were carefully removed before placing a standard absorption medium on the specimen. Then the urine remaining of RIPPING materials was determined by the mass difference in the standard absorption medium (m_2_ − m_1_).

For the test of urine wetback for RIPPING materials, a superabsorbent middle layer with an eight-layer standard absorption medium (spunlaced nonwovens, STT was about 1.7 s) at the bottom was placed on a flat table. Synthetic urine was evenly poured onto the specimen (100 mm × 100 mm) 3 times (5 mL each time, 1 min intervals). After the third pouring of urine, the meshed pore top layer was carefully replaced by a dry counterpart, then a standard absorption medium (m_1_) was placed on the specimen and pressed by briquetting (diameter 100 mm, weight 4 kg). After 1 min of absorption, the mass of the standard absorption medium was determined (m_2_). Therefore, the urine wetback of RIPPING materials was characterized by the mass difference in the standard absorption medium (m_2_ − m_1_).

For the test of wetness of RIPPING materials, a specimen (100 mm × 100 mm) consisting of a superabsorbent layer and a dry meshed pore top layer with an eight-layer standard absorption medium (spunlaced nonwovens, STT was about 1.7 s) at the bottom was placed on a flat table. Synthetic urine was evenly poured onto the specimen 3 times (5 mL each time, 1 min intervals). After the third pouring of urine, a standard absorption medium (m_1_) was placed on the specimen and pressed by briquetting (diameter 100 mm, weight 4 kg). After 1 min of absorption, the mass of the standard absorption medium was determined (m_2_). Therefore, the wetness of RIPPING materials was characterized by the mass difference in the standard absorption medium (m_2_ − m_1_).

Characterization of RIPPING diapers and commercial diapers. To compare the performance of RIPPING diapers with commercial diapers, urine absorption, wetness, urine leakage, STT, and urine run-off were tested according to the abovementioned standards. Particularly, the size of diapers for these tests was 100 mm × 300 mm. For urine absorption, the superabsorbent materials of diapers with a size of 100 mm × 300 mm were dipped into 3500 mL urine for 60 min and weighed before and after absorbing urine. For STT and run-off, 40 mL urine was applied once instead of 3 times (5 mL each time, 1 min intervals). For wetness, urine was applied twice (40 mL each time, 5 min intervals). Urine leakage was measured in a similar way to wetness, but the standard absorption medium was placed under diapers before pouring the urine. The urine leakage was determined by the mass difference in the standard absorption medium before and after pouring urine.

Evaluation of comfort and skin health properties. The stiffness of the top layers was evaluated using a standard stiffness tester (LLY-01, Laizhou Electron Instrument Co., Ltd., Yantai, China) to determine the flexibility of the materials. To assess skin health factors related to diaper dermatitis, skin irritation and relative humidity tests were conducted. Healthy volunteers were asked to wear the RIPPING materials for 4 h, after which the skin condition was visually inspected, and the skin relative humidity was recorded using a moisture meter. Furthermore, the in vitro cytotoxicity of the materials was evaluated using NIH3T3 cells. For the cytotoxicity assay, sample extracts were prepared by incubating the materials in complete DMEM medium at a concentration of 10 mg/mL for 24 h, followed by filtration through a 0.22 μm sterile membrane filter. NIH3T3 cells were seeded (10^5^ cells/well) and allowed to adhere for 12 h. The culture medium was then replaced with the sterile extract solution, and the cells were incubated for an additional 12 h. Cell viability was evaluated using the Cell Counting Kit-8 (CCK-8) assay, and the absorbance was measured at 450 nm.

### 2.4. Statistical Analysis

The values were obtained from at least 3 independent samples per experiment and presented as the mean ± standard deviation. A significant difference was analyzed by performing an unpaired Two-Sample *t*-test and significance was considered when *p* < 0.05.

## 3. Results

### 3.1. Design of Rapid Urine-Penetrating, Self-Drying Diapers

**Figure 1:**
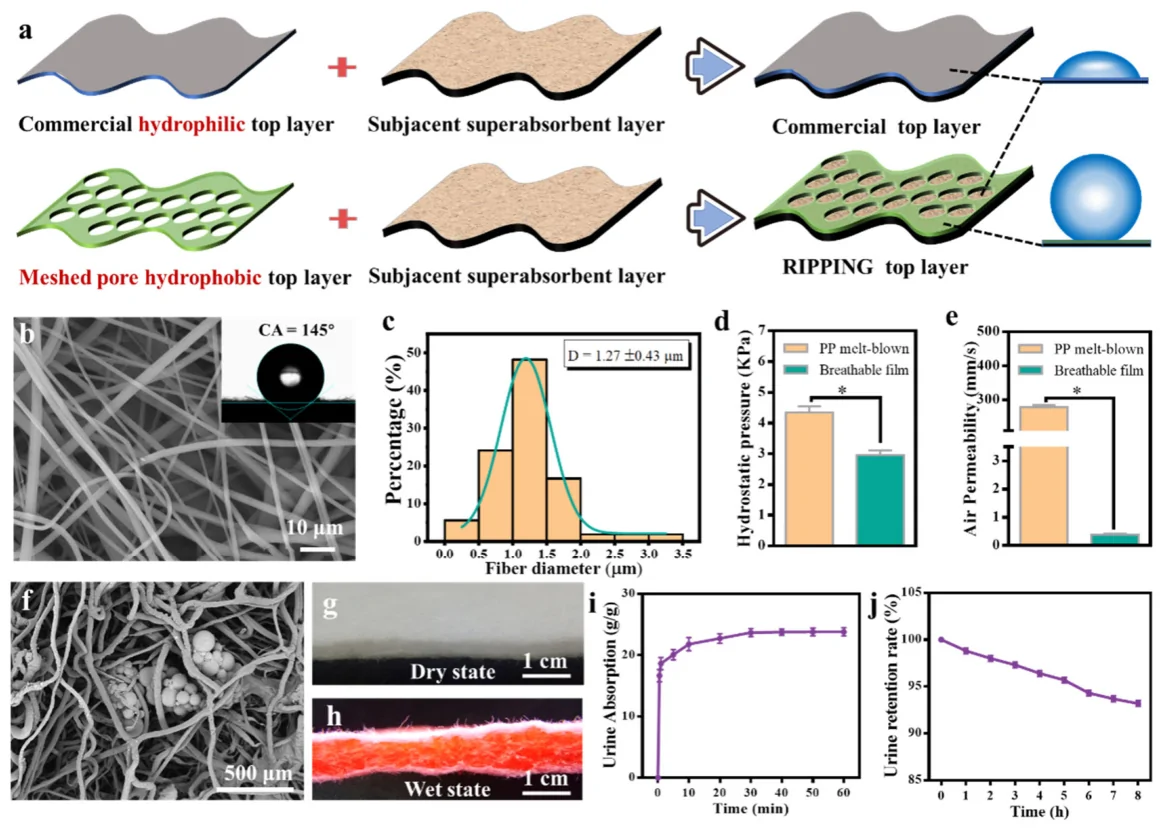
The design of rapid urine-penetrating, self-drying diapers. (**a**) Schematic diagrams showing the design difference between commercial and RIPPING top layer for diapers. (**b**) An SEM image, water contact angle, and (**c**) fiber diameter distribution of PP melt-blown materials. (**d**,**e**) Comparison between PP melt-blown materials and a commercial breathable film: (**d**) hydrostatic pressure, (**e**) air permeability. (**f**) An SEM image of the superabsorbent materials. (**g**,**h**) Optical images of the superabsorbent materials (**g**) before and (**h**) after absorbing urine. (**i**) Urine absorption rate as a function of time under no pressure. (**j**) Urine retention rate as a function of time. The two-tailed Student’s *t*-test was performed, * *p* < 0.05.

Rapid urine penetration and surface self-drying are essential requirements for diapers, but these two conflicting properties can rarely be achieved at the same time. Particularly, a hydrophilic top layer can achieve rapid urine penetration, which becomes wet after urine absorption and fails to maintain a super dry surface for diapers. In contrast, a hydrophobic top layer will prevent urine penetration. To address the abovementioned challenge, we designed rapid urine-penetrating, self-drying materials (RIPPING materials) by making use of the synergy between the meshed pore hydrophobic top layer and superabsorbent middle layer (Figure 1a). In this study, self-drying property is defined as the rapid removal of liquid from the skin-facing surface after fluid insult, characterized by rapid liquid transfer into the absorbent core, low surface liquid retention, and reduced rewetting. We hypothesized that the meshed pores of the hydrophobic top layer would allow for direct urine absorption by the subjacent superabsorbent middle layer. In addition, urine would be minimally retained on the diaper surface by the meshed pore material as it is inherently hydrophobic. Importantly, meshed pores could be properly designed to prevent contact between skin and superabsorbent materials, ensuring a dry surface after urine absorption.

To ensure superior air breathability and hydrophobicity, PP melt-blown materials were selected as the top layer and the back layer. Attributed to the hydrophobic property of PP polymer, the water contact angle of PP melt-blown materials was 145° (Figure 1b). To eliminate the influence of material thickness and weight on the performance comparison, the thickness (0.22 mm) and gram per square meter (GSM, 54.5 g/m^2^) of the PP melt-blown materials were controlled to be comparable to those of the commercial breathable film. Despite the comparable thickness and GSM, the two materials exhibited markedly different microstructures. Unlike the commonly used breathable film (Figure S1a), the PP melt-blown materials consisted of fine fibers with an average diameter of 1.27 ± 0.43 μm (Figure 1c), rendering them with small pores in a diameter of 11.75 ± 3.35 μm (Figure S1b) and superior mechanical properties (Figure S1c–f). It should be noted that the PP melt-blown materials were used as the back layer to prevent urine leakage from the superabsorbent layer, while also ensuring proper breathability. Results indicated that the hydrostatic pressure of PP melt-blown materials was 4.35 ± 0.19 kPa (Figure 1d), which was much higher than that of commercial breathable film (2.96 ± 0.15 kPa). In addition, the air permeability of PP melt-blown materials (279.02 ± 7.21 mm s^−1^) was also much higher than that of commercial breathable film (0.39 ± 0.03 mm s^−1^, Figure 1e), indicating that the use of PP melt-blown materials as the back layer will not only be safe to prevent urine leakage but will also greatly enhance the breathability and thus comfortability of the RIPPING materials for diaper applications.

The superabsorbent layer is the key to absorbing and storing urine. In this study, superabsorbent materials (Figure 1f) containing superabsorbent polymers (SAPs) (Figure S2a) and polyethylene/polypropylene bi-component fibers (Figure S2b) were used as the middle layer. Notably, the SAP spheres can absorb and store urine, while the bi-component fibers can uniformly distribute and stabilize the SAP spheres, ensuring the superabsorbent materials with proper mechanical properties (Figure S2c–f). Attributed to the fixing effect of bi-component fibers, the superabsorbent materials can maintain an integral structure after absorbing urine (Figure 1g,h), although the thickness and area density of the superabsorbent materials dramatically increased (Figure S2g,h). Importantly, the superabsorbent materials exhibited a rapid urine absorption capability of 16.66 ± 1.01 g g^−1^ in 30 s and became saturated at 23.68 ± 0.63 g g^−1^ in 30 min (Figure 1i), which was minimally influenced by applied pressure (Figure S2i). In addition, the superabsorbent materials showed an eminent urine retention rate as a function of time, which decreased slightly from 100% to 93.2% ± 0.29% during the 8 h test (Figure 1j). These results indicate that the selected superabsorbent materials have excellent stability in urine retention, urine absorption rate, and urine absorption capacity, making them suitable for the application of disposal diapers.

### 3.2. Design and Fabrication of the Meshed Pore Melt-Blown Materials

**Figure 2:**
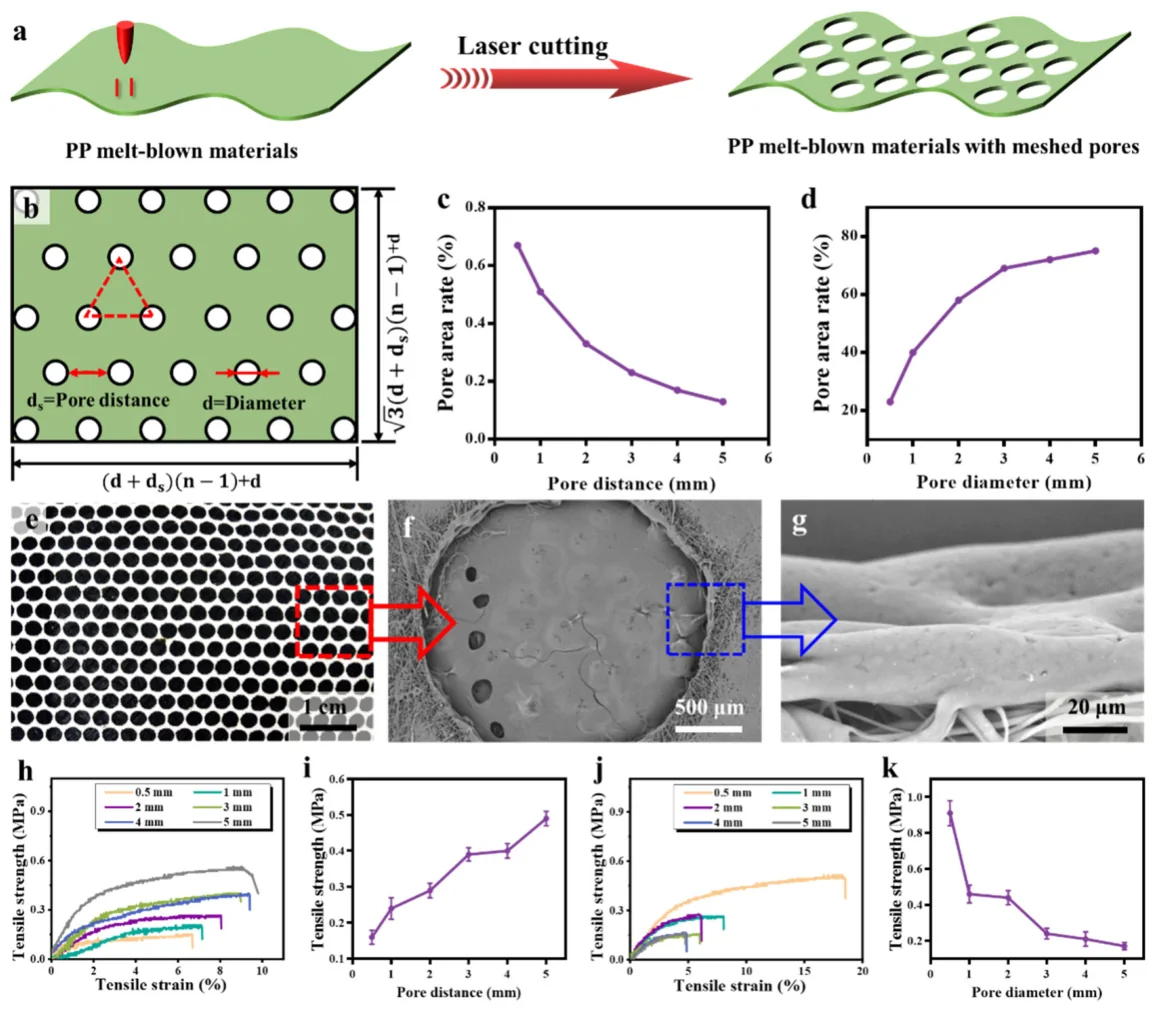
Design and fabrication of the meshed pore melt-blown materials. (**a**) Generation of meshed pores by laser cutting. (**b**) The design of meshed pore pattern, and definition of pore diameter and pore distance. (**c**,**d**) Pore area rate as a function of (**c**) pore distance and (**d**) pore diameter. (**e**) An optical image of the meshed pore materials. (**f**,**g**) SEM images of the meshed pore structure. (**h**–**k**) Mechanical properties of meshed materials with (**h**,**i**) different pore distances with a pore diameter of 3 mm and (**j**,**k**) different pore diameters with a pore distance of 0.5 mm.

To ensure that urine can penetrate through the hydrophobic top layer, the PP melt-blown materials were further processed with a meshed pore structure using a laser cutter (Figure 2a). Considering that the urine droplets tend to be spherical when contacting the hydrophobic top layer, circular meshed pores were generated. We hypothesized that the greater density and bigger diameter of meshed pores would render better urine penetration performance. While preventing direct skin contact with the urine on the surface of the superabsorbent middle layer, the meshed pores should be as small as possible. Therefore, the pattern connecting the centers of three adjacent pores was designed to be an equilateral triangle (Figure 2b), to enable the production of meshed pores with the highest density (Figure 2b). Logically, pore diameter and pore distance were found to be the key parameters for determining the pore area rate.

Based on the abovementioned hypothesis, we produced samples with various pore distances and pore diameters. According to the theoretical calculation, the pore area rate was elevated from 12.8% to 66.7% when the pore distance was decreased from 5 mm to 0.5 mm by keeping the pore diameter constant at 3 mm (Figure 2c and Figure S3). It should be noted that we were not able to generate meshed pores without defects when the pore distance was lower than 0.5 mm. Thus, samples with various pore diameters were generated by keeping the pore distance constant at 0.5 mm. Correspondingly, the pore area rate increased from 21.4% to 74.4% when the pore diameter was enhanced from 0.5 mm to 5 mm (Figure 2d and Figure S4). Our results indicate that the laser cutter can generate well-controlled patterns (Figure 2e), which were in good agreement with the designed ones (Figures S3 and S4). In addition, the edge of meshed pores exhibited bonded fibers, which were melted by a laser (Figure 2f,g), validating the suitability for generating meshed pores using a laser cutter. Although increasing the pore diameter resulted in decreased tensile strength and elongation at break (Figure S5), the selected 3 mm/0.5 mm structure provides a balance between liquid transport, breathability, and mechanical integrity. Notably, the top layer functions primarily as a liquid-permeable interface rather than the main load-bearing component of the diaper, while the integrated multilayer structure of the top layer and the superabsorbent material will ensure enough mechanical stability during use. Nevertheless, the present quasistatic mechanical tests do not fully reproduce the repeated deformation associated with infant movements, and further cyclic loading tests would be valuable for evaluating its long-term dynamic stability.

Furthermore, to address the critical factors influencing diaper dermatitis and overall comfort, we systematically investigated the material properties related to skin health. The stiffness of the RIPPING materials was comparable to that of the commercial diaper, with values of 52.75 ± 2.98 cN·cm and 51.26 ± 3.12 cN·cm, respectively (Figure S6a), indicating that the formation of thermally bonded edges exhibited negligible influence on stiffness, which should be attributed to the narrow width and gradient structure of thermally bonded edges. In terms of biocompatibility, cytotoxicity assays using NIH3T3 cells demonstrated outstanding cell viability for the RIPPING materials (Figure S6b). Notably, no significant difference in cell viability was observed among the RIPPING materials, the commercial diapers, and the DMEM medium. In addition, no erythema or edema was observed on the skin after 4 h of direct contact with the materials (Figure S6c). A key factor in preventing diaper dermatitis is maintaining an appropriate microclimate. Our results showed that the skin relative humidity remained unchanged after 4 h of direct contact with the RIPPING materials, largely attributed to their excellent breathability and the presence of large meshed pores. Conversely, the skin relative humidity associated with the commercial top layer increased from 30.3% to 32.6%. Collectively, these findings provide compelling evidence that the RIPPING materials possess superior comfort and favorable skin health-preserving properties, addressing the fundamental triggers of diaper dermatitis.

### 3.3. Urine Absorption Properties of RIPPING Materials

In general, diapers with self-drying, rapid urine absorption, and high urine absorption capacity are highly desired [32]. To in-depth evaluate the influence of meshed pore structure on urine absorption and self-drying properties, meshed pore melt-blown materials and RIPPING materials were characterized regarding urine remaining, urine wetback, wetness, strike-through time (STT), and urine run-off, which represent residual urine on the surface of the material after absorbing urine, urine wetback from the superabsorbent through meshed pores, total urine interacting with infants’ skin, urine absorption speed, and unabsorbed urine, respectively. It should be noted that the RIPPING materials were made by placing the meshed pore hydrophobic materials on top of the superabsorbent materials.

**Figure 3:**
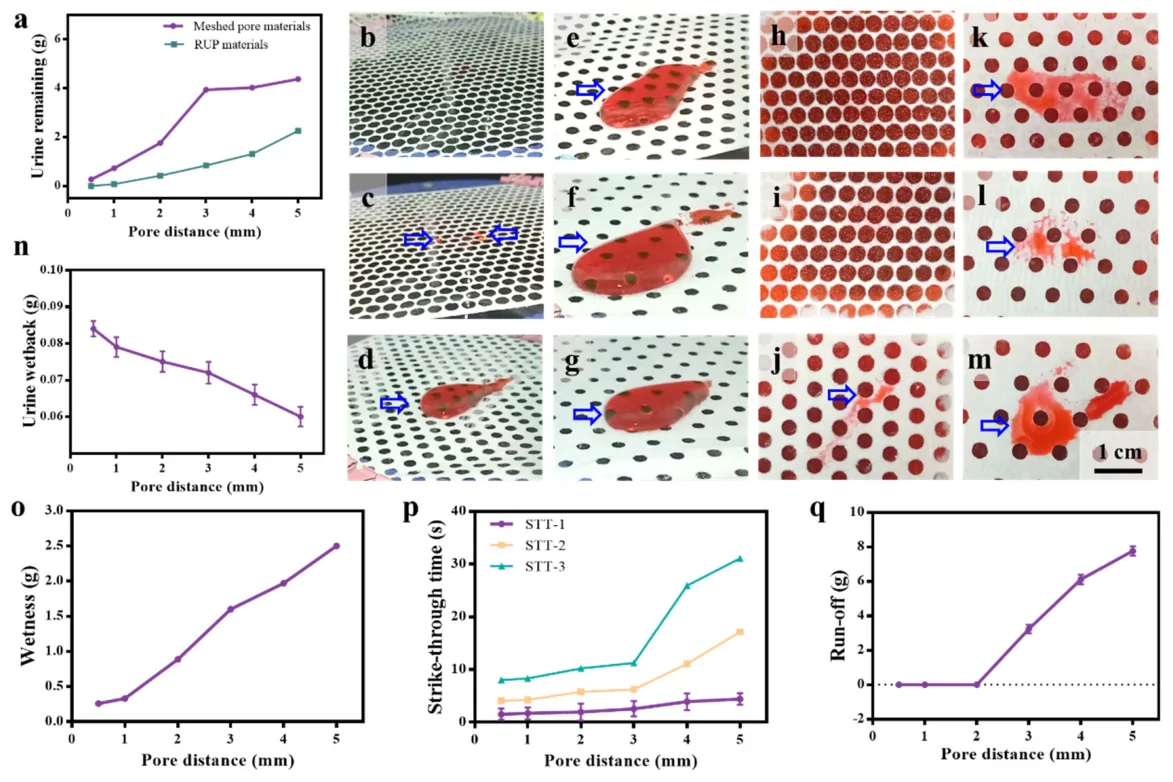
Influence of pore distance on urine absorption properties of RIPPING materials by keeping pore diameter constant at 3 mm. (**a**) Urine remaining. (**b**–**g**) Images of meshed pore materials right after pouring urine: (**b**) 0.5 mm, (**c**) 1 mm, (**d**) 2 mm, (**e**) 3 mm, (**f**) 4 mm, (**g**) 5 mm. (**h**–**m**) Images of RIPPING materials containing meshed pore materials after pouring urine: (**h**) 0.5 mm, (**i**) 1 mm, (**j**) 2 mm, (**k**) 3 mm, (**l**) 4 mm, (**m**) 5 mm. The position indicated by this arrow is the remaining urine. (**n**) Urine wetback. (**o**) Wetness. (**p**) Strike-through time. (**q**) Run-off.

To accurately measure the urine remaining on the meshed pore hydrophobic materials, samples were held in the air with a circular holder when pouring urine on the surface. In this way, urine could penetrate through the meshed pores without being influenced by any contacted materials. By keeping the pore diameter constant at 3 mm, the urine remaining in the meshed pore materials increased from 0.270 ± 0.081 g to 4.380 ± 0.082 g when the pore distance was elevated from 0.5 mm to 5 mm (Figure 3a). Notably, obvious urine was observed on the surface of the meshed pore materials when the pore distance was equal to or larger than 1 mm (Figure 3b–g and Movie S1), indicating that a larger area of hydrophobic materials can provide more resistance for urine penetration (Figure 1c).

To obtain the urine remaining on the RIPPING materials, the superabsorbent layer of the RIPPING materials was carefully removed after pouring urine on the surface. Results indicated that the urine remaining on the RIPPING materials grew from 0 g to 2.260 ± 0.060 g (Figure 3a) and the urine remaining was visually observed when the pore distance was equal to and larger than 2 mm (Figure 3h–m and Movie S2). It is worth noting that the urine remaining of the RIPPING materials was significantly less than that of the respective meshed pore materials at all different pore distances investigated (Figure 3b–m and Movies S1 and S2), indicating that the addition of the superabsorbent materials could help enhance the urine penetration.

To measure the urine wetback of RIPPING materials, the meshed pore top layer was carefully replaced with a dry counterpart after pouring urine on the surface. Results showed that urine wetback slightly decreased from 0.084 ± 0.002 g to 0.060 ± 0.003 g when the pore distance was increased from 0.5 mm to 5 mm (Figure 3n), which should be ascribed to the reduced pore area rate and thus less contact chance between the standard absorption medium (or human skin for practical use) and the superabsorbent middle layer. It should be noted that urine wetback obtained from the experiment was less than the real scenario as the replaced dry counterpart would inevitably consume some urine.

As the synergetic effect of urine wetback and urine remaining, the wetness of RIPPING materials was enhanced from 0.260 ± 0.012 g to 2.500 ± 0.015 g when the pore distance was increased from 0.5 mm to 5 mm, indicating that smaller pore distance of meshed pore top layer enabled RIPPING materials to have a better self-drying property (Figure 3o, which can also be validated by the visual observation (Figure 3h–m and Movie S2). It could be found that the wetness of RIPPING materials was mainly determined by the urine remaining especially when the pore distance was large. Importantly, STT results further showed that fast urine absorption was achieved when the pore distance was less than or equal to 3 mm (Figure 3p). Moreover, urine run-off remained at 0 g when the pore distance was less than or equal to 3 mm, and then increased to 7.768 ± 0.272 g when the pore size was 5 mm, further validating that a smaller pore distance endowed RIPPING materials with a better urine absorption property (Figure 3q).

**Figure 4:**
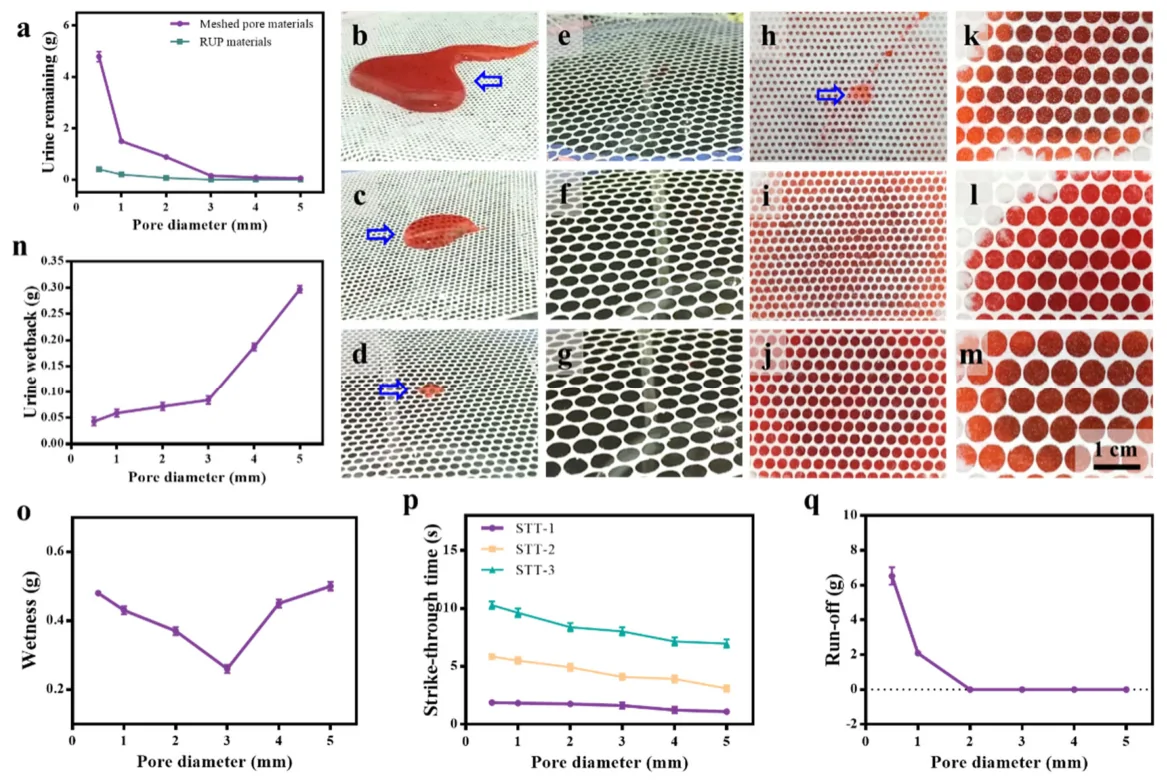
Influence of pore diameter on urine absorption properties of RIPPING materials by keeping pore distance constant at 0.5 mm. (**a**) Urine remaining. (**b**–**g**) Images of meshed pore materials right after pouring urine: (**b**) 0.5 mm, (**c**) 1 mm, (**d**) 2 mm, (**e**) 3 mm, (**f**) 4 mm, (**g**) 5 mm. (**h**–**m**) Images of RIPPING materials containing meshed pore materials after pouring urine: (**h**) 0.5 mm, (**i**) 1 mm, (**j**) 2 mm, (**k**) 3 mm, (**l**) 4 mm, (**m**) 5 mm. The position indicated by this arrow is the remaining urine. (**n**) Urine wetback. (**o**) Wetness. (**p**) Strike-through time. (**q**) Urine run-off.

Based on the abovementioned results, we further investigated whether optimization of pore diameter can improve the urine absorption property while keeping the pore distance at 0.5 mm. When pore diameter was increased from 0.5 mm to 5 mm, the urine remaining decreased from 0.40 ± 0.01 g to 0 g and from 4.80 ± 0.20 g to 0.05 ± 0.01 g for RIPPING materials and meshed pore materials, respectively (Figure 4a). The critical pore diameter for no obvious urine remaining was 0.5 mm and 3 mm for RIPPING materials and meshed pore materials, respectively (Figure 4b–m, Movies S3 and S4). In contrast, the urine wetback of RIPPING materials increased from 0.043 ± 0.008 g to 0.297 ± 0.007 g (Figure 4n), indicating that a larger pore diameter can significantly increase the direct contact possibility between the superabsorbent materials and the standard absorption medium.

Attributed to the synergetic effect of urine remaining and urine wetback, the lowest wetness of RIPPING materials (0.260 ± 0.012 g) was achieved when the pore diameter of the meshed pore materials was 3 mm. Importantly, both larger and smaller pore diameters of the meshed pore materials led to a drastic increase in wetness (Figure 4o). According to the results of urine remaining, urine wetback, and wetness, the sum of urine remaining and urine wetback aligned well with the real value of wetness. Particularly, urine can rarely penetrate through the small pores of hydrophobic materials when the pore diameter of the meshed pore materials is smaller than 3 mm; therefore, the urine remaining mainly contributed to the elevated wetness. When the pore size was larger than 3 mm, the significantly increased possibility between the superabsorbent materials and the standard absorption medium contributed to the elevated urine wetback, which should be the main reason for the increased wetness. In addition, STT results showed that the RIPPING materials have an excellent urine absorption property, and the STT was decreased by increasing the pore diameter (Figure 4p). The urine run-off results suggested that the superior urine absorption property of RIPPING materials could be achieved when the pore diameter was equal to or larger than 2 mm (Figure 4q). Considering all the results, a pore size of 3 mm and a pore distance of 0.5 mm were considered the best for the following studies to fabricate diapers with self-drying and rapid urine absorption properties. Collectively, these quantitative evaluations provide robust data confirming the surface self-drying performance of the optimized RIPPING materials. By maintaining zero urine remaining and a minimal surface wetness of 0.260 ± 0.012 g, the meshed hydrophobic top layer ensures that liquid is instantly cleared into the underlying absorbent core without leaving residual wetness on the skin-contacting interface.

### 3.4. Comparison Between RIPPING Diapers and Commercial Diapers

**Figure 5:**
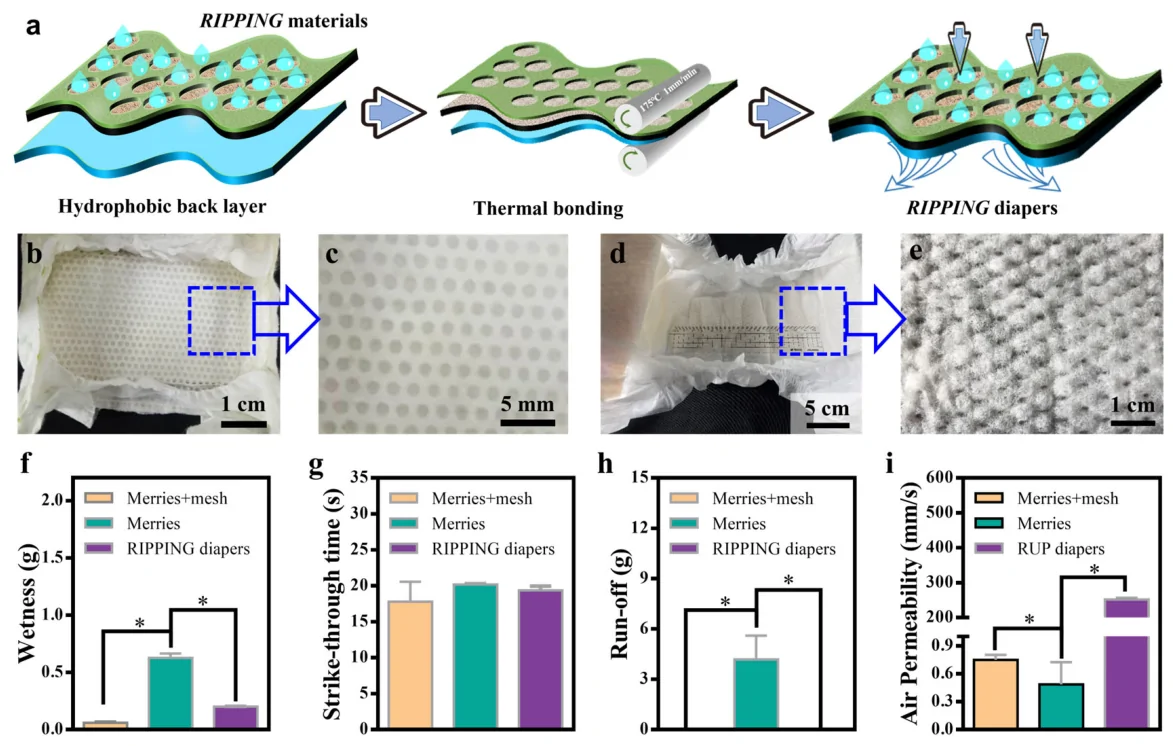
Comparison between RIPPING diapers and commercial diapers. (**a**) Design and fabrication of RIPPING diapers. (**b**) Images of the RIPPING diapers and (**c**) the meshed pore structure. (**d**) Images of the Merries diapers and (**e**) the top layer structure. (**f**) Wetness. (**g**) Strike-through time. (**h**) Urine run-off. (**i**) Air permeability. The two-tailed Student’s *t*-test was performed, * *p* < 0.05.

To verify the performance of RIPPING materials for the application of diapers, RIPPING diapers were manufactured by thermal bonding of the meshed pore hydrophobic top layer, the superabsorbent middle layer, and the hydrophobic back layer (Figure 5a–c). In addition, the most popular Merries diapers with a similar sandwich structure were selected as the control (Figure 5d,e). Interestingly, the wetness of RIPPING diapers (0.20 ± 0.01 g) was significantly lower than that of Merries diapers (0.62 ± 0.04 g), suggesting that the RIPPING materials hold great potential to improve the self-drying properties of diapers.

Recognizing that direct comparison between whole diapers might be influenced by differences in superabsorbent core composition and structural thickness, a Merries + mesh control group was specifically designed to establish a rigorous single-variable comparison. In this group, the commercial top layer of the Merries diaper was replaced with our optimized meshed hydrophobic layer while maintaining the original commercial absorbent core and structural assembly intact. In this way, the effect of meshed pore hydrophobic materials could be verified by comparing them with Merries diapers. Results indicated that the wetness of Merries + mesh diapers (0.06 ± 0.01 g) was even lower than that of RIPPING diapers, verifying that the employment of meshed pore hydrophobic materials could endow diapers with a much drier surface (Figure 5f). This ultra-low wetness of Merries + mesh is attributed to the commercial Merries absorbent core, which features an industrially engineered composite of high-density fluff pulp and SAPs with exceptional localized capillary suction, rapidly drawing liquid away from the interface and locking it tightly. Nevertheless, the present wetback evaluation was conducted under static conditions and did not reproduce the repeated deformation associated with infant movements. Future studies will further investigate the wetback behavior and structural stability of the RIPPING diaper under dynamic cyclic mechanical loading. Although there was no significant difference when measuring STT (Figure 5g), both Merries + mesh and RIPPING diapers have better urine absorption properties, confirmed by the urine run-off test (Figure 5h), which should be attributed to the use of a meshed pore top layer.

Benefiting from the superior hydrostatic pressure resistance and high breathability of the PP melt-blown back layer (Figure 1b–e), the complete RIPPING diaper system achieved an overall air permeability of 251.910 ± 2.850 mm s^−1^, which is over 300 times higher than that of the Merries + mesh system (0.748 ± 0.056 mm s^−1^, Figure 5i). Thus, the complete RIPPING system enables rapid dissipation of microclimate heat and humidity, offering superior wearing comfort. Importantly, the improved air permeability does not lead to more urine leakage. To this end, our results indicate that RIPPING materials are superior for diapers, which can dramatically improve the self-drying property, urine absorption, and air permeability.

## 4. Conclusions

In conclusion, we have developed RIPPING materials with rapid urine-penetrating, self-drying, and breathable properties for the application of diapers. These unique properties were achieved by the design of the RIPPING materials, consisting of a meshed pore hydrophobic top layer, a superabsorbent middle layer, and a hydrophobic back layer. Results indicate that RIPPING materials could greatly improve the comfort of diapers attributed to the breathable and self-drying properties, and thus decrease the prevalence rate of diaper dermatitis. In addition, the RIPPING materials may also be useful for adult incontinence products, including pants and pads and female sanitary napkins.

## Data Availability

The data presented in this study are available on request from the corresponding author.

## Supplementary Materials

The following supporting information can be downloaded at: https://www.mdpi.com/article/10.3390/polym18192383/s1, Figure S1: Comparison between PP melt-blown materials and a commercial breathable film. (a) SEM image of the commercial breathable film. (b) Pore size distribution of PP melt-blown materials. (c) Tensile strength as a function of tensile strain. (d) Tensile strength. (e) Young’s modulus. (f) Tensile strain; Figure S2: Properties of the superabsorbent materials. (a-b) SEM images showing (a) SAPs and (b) Polyethylene/polypropylene bi-component fibers in the superabsorbent materials. (c-f) Properties of superabsorbent materials before and after absorbing urine: (c) Tensile strength as a function of tensile strain, (d) Tensile strength, (e) Young’s modulus, (f) Tensile strain, (g) Thickness, (h) Area density. (i) Saturated urine absorption as a function of pressure; Figure S3: Images of meshed pore materials with different pore distances when the pore diameter was kept constant at 3 mm. (a) 0.5 mm. (b) 1.0 mm. (c) 2.0 mm. (d) 3.0 mm. (e) 4.0 mm. (f) 5.0 mm; Figure S4: Images of meshed pore materials with different pore diameters when the pore distance was kept constant at 0.5 mm. (a) 0.5 mm. (b) 1.0 mm. (c) 2.0 mm. (d) 3.0 mm. (e) 4.0 mm. (f) 5.0 mm; Figure S5: Mechanical properties of meshed materials. (a-b) Different pore distances with a pore diameter of 3 mm. (c-d) Different pore diameters with a pore distance of 0.5 mm; Figure S6: Evaluation of skin-contact safety and comfort-related properties of the RIPPING material. (a) Stiffness. (b) Cytotoxicity evaluation. (c) Representative images after contact with the RIPPING material; Movie S1: Urine absorption of meshed pore materials with different pore distances. (a) 0.5 mm, (b) 1 mm, (c) 2 mm, (d) 3 mm, (e) 4 mm, and (f) 5 mm. Pore diameter was 3 mm; Movie S2: Urine absorption of RIPPING materials containing meshed pore materials with different pore distances. (a) 0.5 mm, (b) 1 mm, (c) 2 mm, (d) 3 mm, (e) 4 mm, and (f) 5 mm. Pore diameter was 3 mm; Movie S3: Urine absorption of meshed pore materials with different pore diameters. (a) 0.5 mm, (b) 1 mm, (c) 2 mm, (d) 3 mm, (e) 4 mm, and (f) 5 mm. Pore distance was 0.5 mm; Movie S4. Urine absorption of RIPPING materials containing meshed pore materials with different pore diameters. (a) 0.5 mm, (b) 1 mm, (c) 2 mm, (d) 3 mm, (e) 4 mm, and (f) 5 mm. Pore distance was 0.5 mm.
