# Supplementary material for: Developing Rapid Urine-Penetrating, Self-Drying, and Breathable Materials for Disposal Diapers
Source: Polymers (Basel). 2026 Sep 29;18(19):2383. doi: 10.3390/polym18192383 (PMC13666657; doi:10.3390/polym18192383)
Supplement: Supplementary file 1 [file polymers-18-02383-s001.zip › polymers-4539818-supplementary.pdf]

Supporting information for

Developing Rapid Urine Penetrating, Self-Drying, and Breathable Materials for Disposal Diapers

Yuanduo Wang [a,1], Dan Wang [a,1], Yuxiao Wang [1], Mengyao Ruan [1], Xiangyu Jin [1], Andreii S. Kritchenkov [2,3], Gilson Khang [4], Sergei V. Kostjuk [5,6], Jiansheng Guo [1,*], Wanjun Liu [1,*]

1. Shanghai Frontiers Science Center of Advanced Textiles, College of Textiles, Donghua University, Shanghai, 201620, China
2. Peoples' Friendship University of Russia (RUDN University), Moscow 117198, Russian Federation
3. Institute of Technical Acoustics NAS of Belarus, Vitebsk 210009, Republic of Belarus
4. Department of Bionanotechnology and Bio-Convergence Engineering, Jeonbuk National University, Jeonbuk 54896, Republic of Korea
5. Department of Chemistry, Belarusian State University, Minsk 220006, Belarus
6. Sorbonne Universite, CNRS, Institut Parisien de Chimie Moleculaire, Equipe Chimie des Polymeres, 4 place Jussieu, 75252 Paris Cedex 05, France

* Correspondence: jsguo@dhu.edu.cn (J.G.); wjliuok@dhu.edu.cn (W. L.)

[a] These authors contributed equally to this work.

$$S_1 = \frac{1}{4}\pi d^2\left(n^2 + (n-1)^2\right) = \frac{1}{4}\pi d^2(2n^2 - 2n + 1) \quad \text{(S1)}$$

$$S = \sqrt{3}(n-1)^2{(d+d_s)}^2 + 2\sqrt{3}(d+d_s)(n-1) + d^2 \quad \text{(S2)}$$

$$R = \frac{S_1}{S} = \frac{\sqrt{3}\pi d^2}{6(d+d_s)^2} \quad \text{(S3)}$$

Here, S_1, d, n, S, d_s, and R represent pore area, pore diameter, pore number on the edge, total area, pore distance, and pore area rate, respectively.

When n approaches infinity, the pore area rate (R) can be expressed as follows:

$$R = \frac{S_1}{S} = \frac{\sqrt{3}\pi d^2}{6(d+d_s)^2} \quad \text{(S4)}$$

When the pore diameter is 3 mm, the pore area rate can be expressed as follows:

$$R = \frac{3\sqrt{3}\pi}{2(3+d_s)^2} = \frac{8.16}{(3+d_s)^2} \quad \text{(S5)}$$

When the pore distance is 0.5 mm, the pore area rate can be expressed as follows:

$$R = \frac{\sqrt{3}\pi d^2}{6(d+0.5)^2} = \frac{0.91d^2}{(d+0.5)^2} \quad \text{(S6)}$$

Our results indicate that the superabsorbent materials could stay integral with the help of the bi-component fibers (**Figure 1f-h, S2a-b**), although the mechanical properties were weakened after absorbing urine (**Figure S2c**). In brief, the tensile strength and Young's modulus decreased from 0.35 MPa to 0.08

MPa and from 1.35 ± 0.02 MPa to 0.27 ± 0.01 MPa, respectively (**Figure S2d-e**). While the elongation at break was elevated from 24.77 % to 31.15 % (**Figure S2f**). After absorbing urine, the thickness of the superabsorbent materials grew from 2.44 ± 0.29 mm to 6.74 ± 0.31 mm (**Figure S2g**) due to the swelling of SAPs and the area density increased from 350 ± 15 g m^{-2} to 8570 ± 270 g m^{-2} (**Figure S2h**). It should be noted that synthetic urine was used for all experiments[25].

Next, we investigate the urine absorption properties of the superabsorbent materials. Under no pressure, the urine absorption increased dramatically from 0 g g^{-1} to 16.66 ± 1.01 g g^{-1} in 30s, then slowed down and became saturated at 23.68 ± 0.63 g g^{-1} in 30 minutes (**Figure 1i**), showing a rapid urine absorption property of the superabsorbent materials. In addition, pressure was applied to simulate the state of the diaper during the practical scenario. Specifically, when the pressure was enhanced from 0 to 4900 Pa, the saturated urine absorption slightly reduced from 23.79 ± 0.68 g g^{-1} to 18.43 ± 0.06 g g^{-1} (**Figure S2i**). In addition, the superabsorbent materials also showed an eminent urine retention rate as a function of time, which decreased slightly from 100% to 93.2% ± 0.29% when time flew from 0 h to 8 h (**Figure 1j**). These results indicate that the selected superabsorbent materials have excellent stability of urine retention, urine absorption rate, and urine absorption capacity, making them suitable for the application of disposal diapers.

In addition, we investigated the influence of the pore structure on the mechanical properties of PP melt-blown materials. In brief, the mechanical properties were enhanced with the increase of pore distance (**Figure 2h**). Specifically, the tensile strength, tensile strain and Young's modulus grow from 0.16 ± 0.02 MPa to 0.49 ± 0.02 MPa, from 7.40% ± 1.33% to 19.51% ± 1.13%, and from 2.98 ± 0.26 MPa to 8.31 ± 0.31 MPa when the pore distance is elevated from 0.5 mm to 5 mm (**Figure 2i, S5a-b**). On the contrary, the mechanical properties of meshed materials were weakened with the increase of pore diameter (**Figure 2j-k, S5c-d**). Therefore, we can conclude that the mechanics of the meshed pore materials were weakened with the increase of pore area rate.

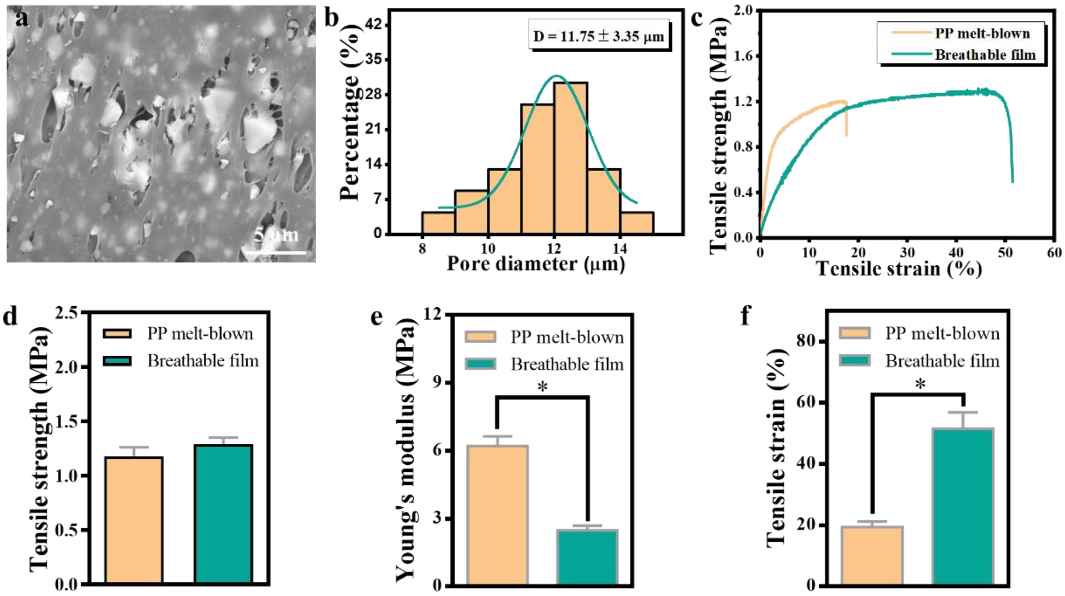

Figure S1. Comparison between PP melt-blown materials and a commercial breathable film. (a) SEM image of the commercial breathable film. (b) Pore size distribution of PP melt-blown materials. (c) Tensile strength as a function of tensile strain. (d) Tensile strength. (e) Young's modulus. (f) Tensile strain.

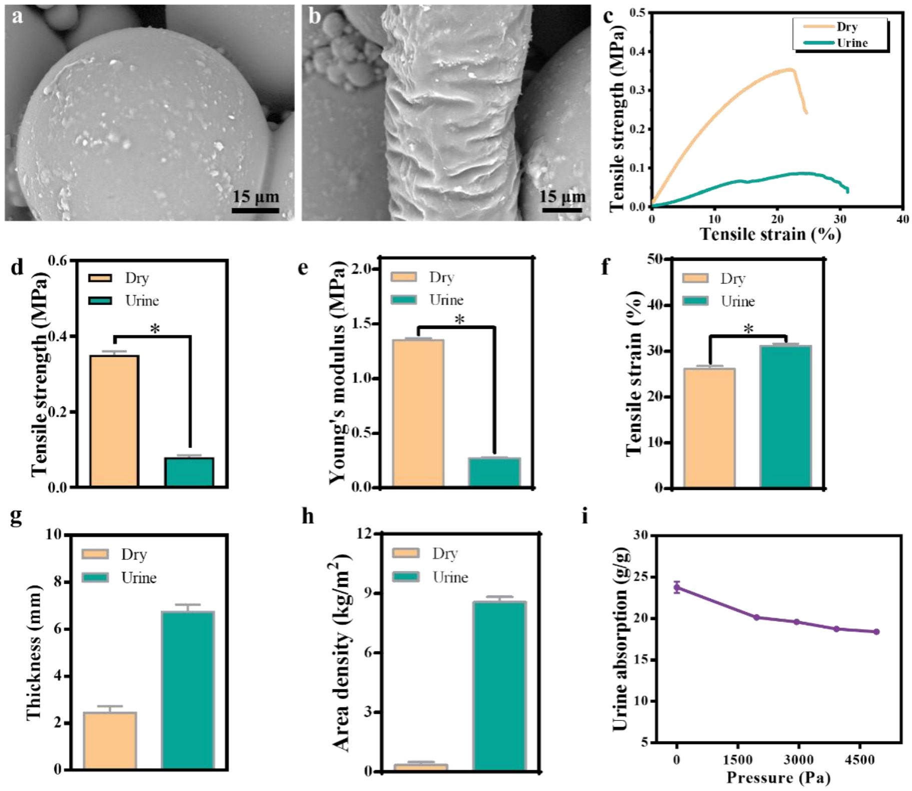

Figure S2. Properties of the superabsorbent materials. (a-b) SEM images showing (a) SAPs and (b) Polyethylene/polypropylene bi-component fibers in the superabsorbent materials. (c-f) Properties of

superabsorbent materials before and after absorbing urine: (c) Tensile strength as a function of tensile strain, (d) Tensile strength, (e) Young's modulus, (f) Tensile strain, (g) Thickness, (h) Area density. (i) Saturated urine absorption as a function of pressure.

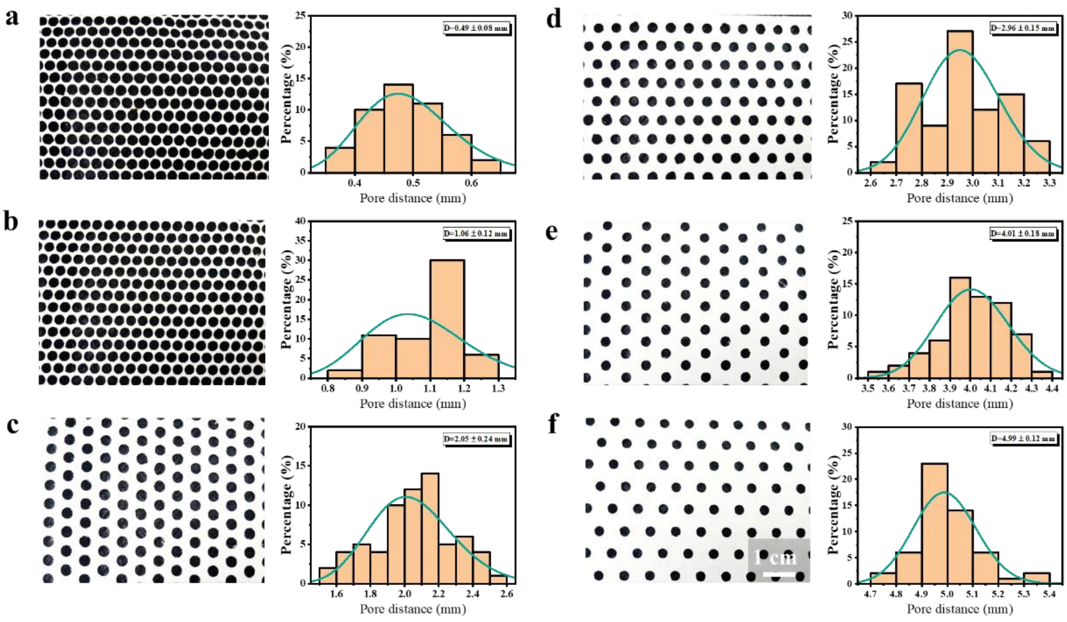

Figure S3. Images of meshed pore materials with different pore distances when the pore diameter was kept constant at 3 mm. (a) 0.5 mm. (b) 1.0 mm. (c) 2.0 mm. (d) 3.0 mm. (e) 4.0 mm. (f) 5.0 mm.

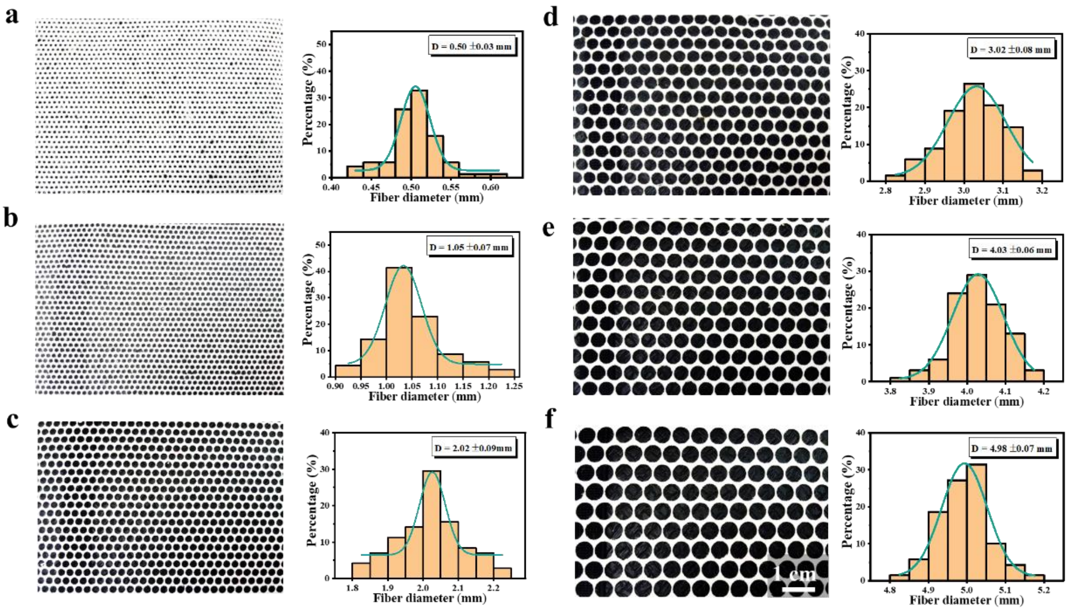

Figure S4. Images of meshed pore materials with different pore diameters when the pore distance was kept constant at 0.5 mm. (a) 0.5 mm. (b) 1.0 mm. (c) 2.0 mm. (d) 3.0 mm. (e) 4.0 mm. (f) 5.0 mm.

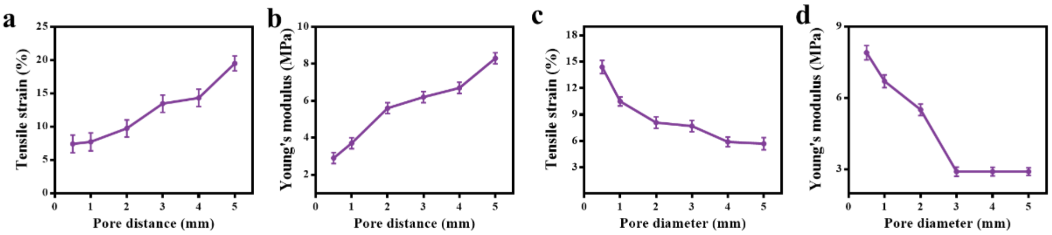

Figure S5. Mechanical properties of meshed materials. (a-b) Different pore distances with a pore diameter of 3 mm. (c-d) Different pore diameters with a pore distance of 0.5 mm.

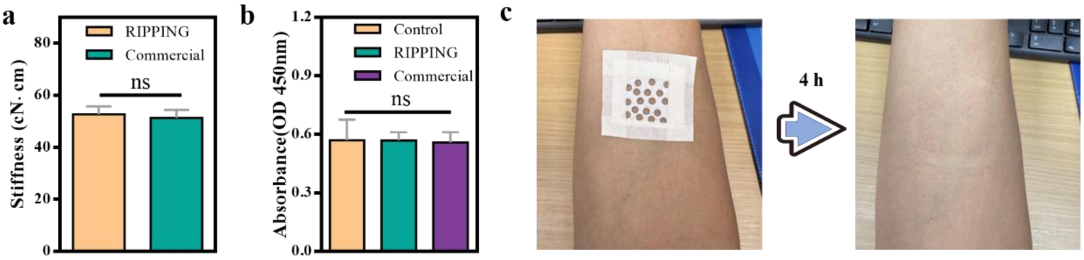

Figure S6. Evaluation of skin-contact safety and comfort-related properties of the RIPPING material. (a) Stiffness. (b) Cytotoxicity evaluation. (c) Representative images after contact with the RIPPING material.

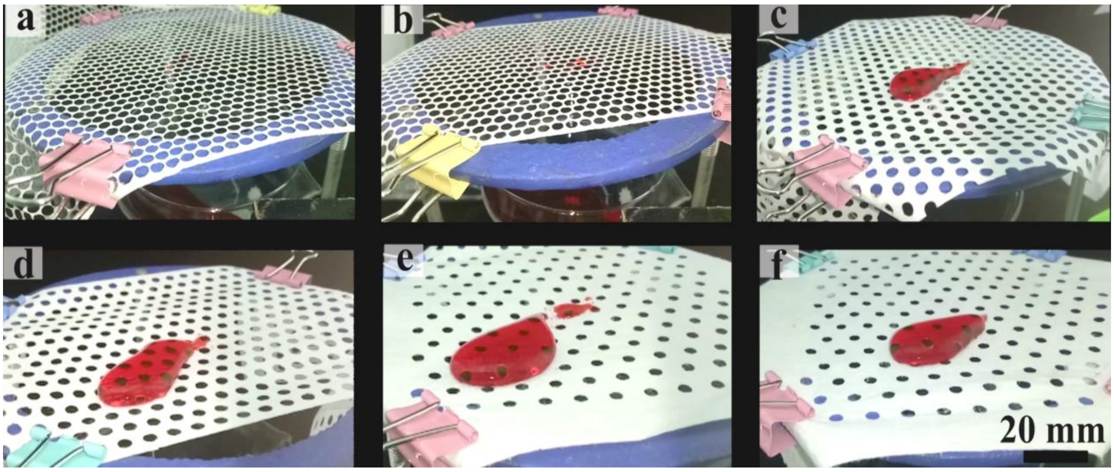

Movie S1. Urine absorption of meshed pore materials with different pore distances. (a) 0.5 mm, (b) 1 mm, (c) 2 mm, (d) 3 mm, (e) 4 mm, and (f) 5 mm. Pore diameter was 3 mm.

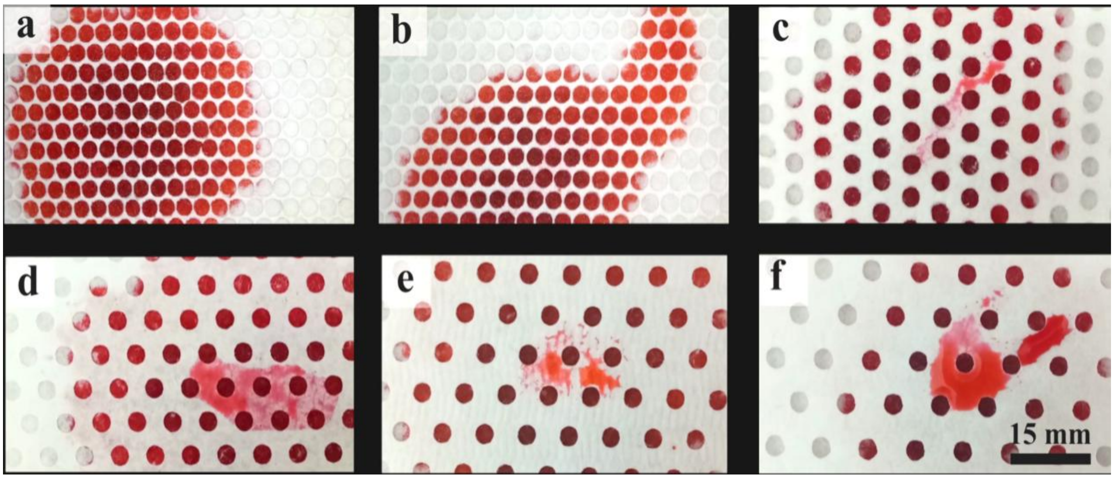

Movie S2. Urine absorption of RIPPING materials containing meshed pore materials with different pore distances. (a) 0.5 mm, (b) 1 mm, (c) 2 mm, (d) 3 mm, (e) 4 mm, and (f) 5 mm. Pore diameter was 3 mm.

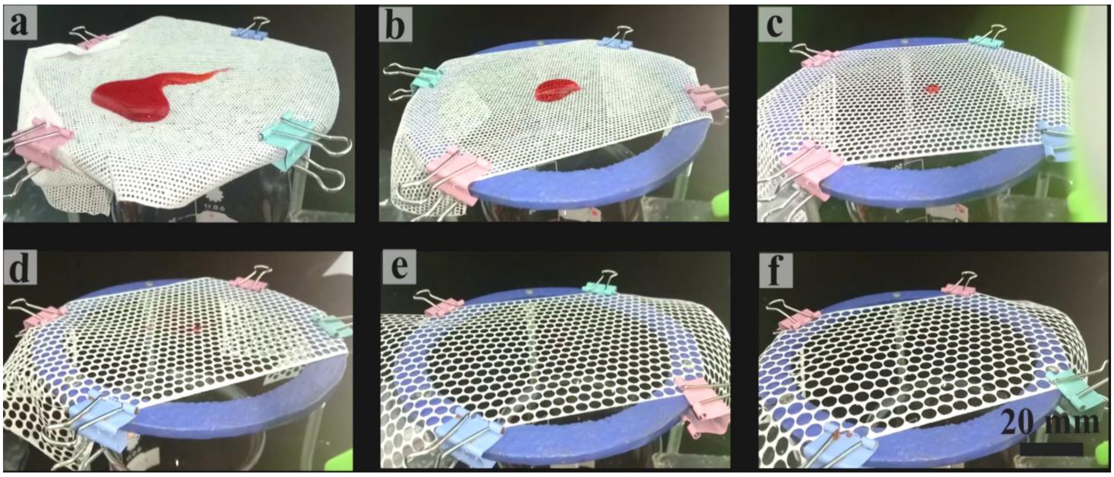

Movie S3. Urine absorption of meshed pore materials with different pore diameters. (a) 0.5 mm, (b) 1 mm, (c) 2 mm, (d) 3 mm, (e) 4 mm, and (f) 5 mm. Pore distance was 0.5 mm.

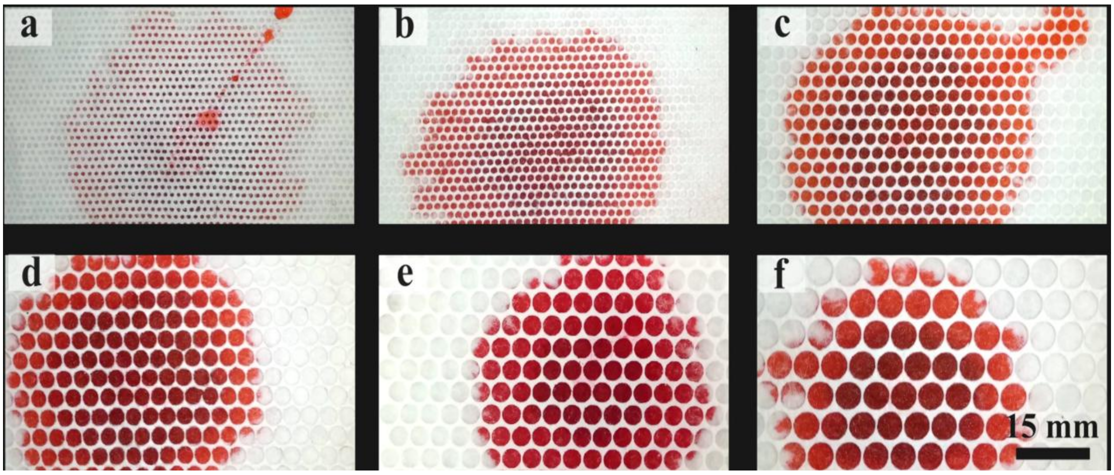

Movie S4. Urine absorption of RIPPING materials containing meshed pore materials with different pore diameters. (a) 0.5 mm, (b) 1 mm, (c) 2 mm, (d) 3 mm, (e) 4 mm, and (f) 5 mm. Pore distance was 0.5 mm.
